# Insights into Exosome Transport through the Blood–Brain Barrier and the Potential Therapeutical Applications in Brain Diseases

**DOI:** 10.3390/ph16040571

**Published:** 2023-04-10

**Authors:** Manal Abdelsalam, Munazza Ahmed, Zaynab Osaid, Rifat Hamoudi, Rania Harati

**Affiliations:** 1 Department of Pharmacy Practice and Pharmacotherapeutics, College of Pharmacy, University of Sharjah, Sharjah P.O. Box 27272, United Arab Emirates; mabdelsalam@sharjah.ac.ae (M.A.); u21104594@sharjah.ac.ae (M.A.); u20105690@sharjah.ac.ae (Z.O.); 2 Research Institute for Medical and Health Sciences, University of Sharjah, Sharjah P.O. Box 27272, United Arab Emirates; 3 Clinical Sciences Department, College of Medicine, University of Sharjah, Sharjah P.O. Box 27272, United Arab Emirates; rhamoudi@sharjah.ac.ae; 4 Division of Surgery and Interventional Science, University College London, London W1W 7EJ, UK

**Keywords:** blood–brain barrier (BBB), neurovascular unit (NVU), exosomes, drug delivery

## Abstract

Drug delivery to the central nervous system (CNS) is limited due to the presence of the blood–brain barrier (BBB), a selective physiological barrier located at the brain microvessels that regulates the flow of cells, molecules and ions between the blood and the brain. Exosomes are nanosized extracellular vesicles expressed by all cell types and that function as cargos, allowing for communication between the cells. The exosomes were shown to cross or regulate the BBB in healthy and disease conditions. However, the mechanistic pathways by which exosomes cross the BBB have not been fully elucidated yet. In this review, we explore the transport mechanisms of exosomes through the BBB. A large body of evidence suggests that exosome transport through the BBB occurs primarily through transcytosis. The transcytosis mechanisms are influenced by several regulators. Inflammation and metastasis also enhance exosome trafficking across the BBB. We also shed light on the therapeutical applications of exosomes for treating brain diseases. Further investigations are essential to provide clearer insights related to trafficking of exosomes across the BBB and disease treatment.

## 1. Introduction

Despite the advancements made in therapeutic delivery, efficient drug delivery to the brain for the treatment of central nervous system diseases remains challenging [1,2,3]. Drug delivery to the CNS is limited due to the presence of the blood–brain barrier (BBB) [4]. BBB is a selective barrier that protects the brain by restricting the entry of potentially neurotoxic molecules [4]. Exosomes are small extracellular vesicles released by all cell types where they function as cargo for communication between cells. Exosomes can cross the BBB; however, their transport mechanism across the barrier remains to be fully understood [5]. In this review, we summarize the current knowledge about the transport mechanisms of exosomes at the BBB and their potential therapeutic role in delivering drugs to the brain. A better understanding of the transport of exosomes through the BBB will aid in developing better therapeutic strategies to treat a wide range of brain diseases [6,7,8,9].

The blood–brain barrier is a selective semi-permeable physiologic membrane located at the brain microvessels, at the interface between the blood and the brain. The BBB regulates the passage of micro- and macromolecules depending on the physiological needs of the brain [9]. A properly functioning BBB protects the neuronal environment, which is necessary to meet metabolic demands and to maintain homeostatic conditions in the brain to sustain neuronal integrity [6,10]. Structurally, the BBB is a muti-cellular structure that consists of the brain endothelial cells tightly attached by inter-endothelial junctional complexes; pericytes; a basement membrane; and astrocytic endfeet [11,12,13]. The brain endothelial cells (BECs) are the key elements forming the wall of the barrier, although their interaction with the other components is essential for inducing and maintaining the barrier’s properties. These BECs are attached by junctional complexes comprising tight junctions, gap junctions and adherent junctions [13,14] that limit the paracellular movement of substances [15,16,17], allowing for entry of specific molecules based on the physiological needs of the brain [6].

Exosomes are nanosized (30–150 nm), circular, or cup-shaped extracellular vesicles that are produced by virtually all normal and pathological cells from the endosomal space, mirroring their originating parent cells. Exosomes carry cell-specific cargo of proteins, lipids, and genetic materials and are of particular interest in scientific research, as they play a major role in inter-cellular communication [5]. Exosomes have been shown to be produced by a variety of cells in the brain, including neurons, microglia, and astrocytes, and they may also be found in the cerebrospinal fluid [18]. They play a pivotal role in cell-to-cell communication, neurogenesis, neuronal stress response, and synaptic plasticity [8,19]. Moreover, they can serve as signals that stimulate the immune system upon a CNS injury [8]. Exosome release is regulated by many factors, including cellular stress, heat, intracellular calcium concentration, phosphatidylinositol 3-kinsase and pH [20].

## 2. Biogenesis and Content of Exosomes

### 2.1. Background

**Figure 1:**
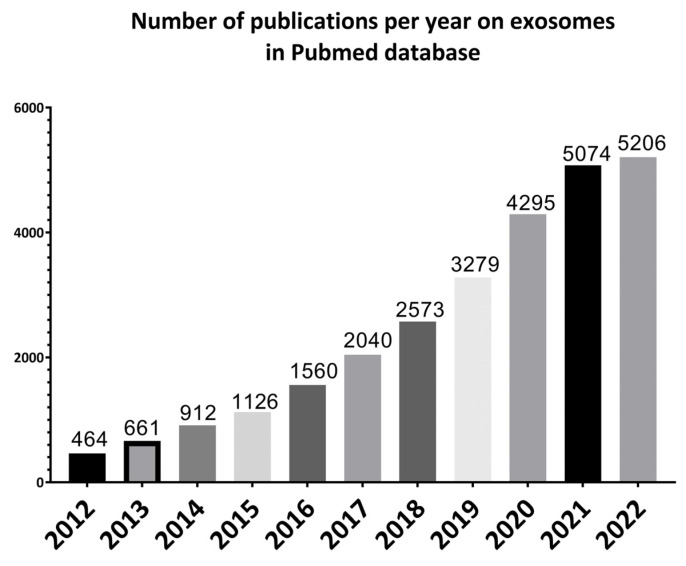
Illustrative bar charts representing the number of papers published per year on exosomes for the past 10 years from 2012 to 2022 using PubMed online database.

Exosomes were first discovered by Stahl and Johnstone groups in 1983 in maturing mammalian reticulocytes. The term “exosomes” was coined in 1987, even though the term was used a few years earlier to describe different membrane segments that were isolated from biological fluids. Since then, a growing number of studies have been performed on exosomes (Figure 1) [21] because of their important role and potential in understanding the pathogenesis as well as treatment and diagnosis of diseases [22].

### 2.2. Exosomes Biogenesis

**Figure 2:**
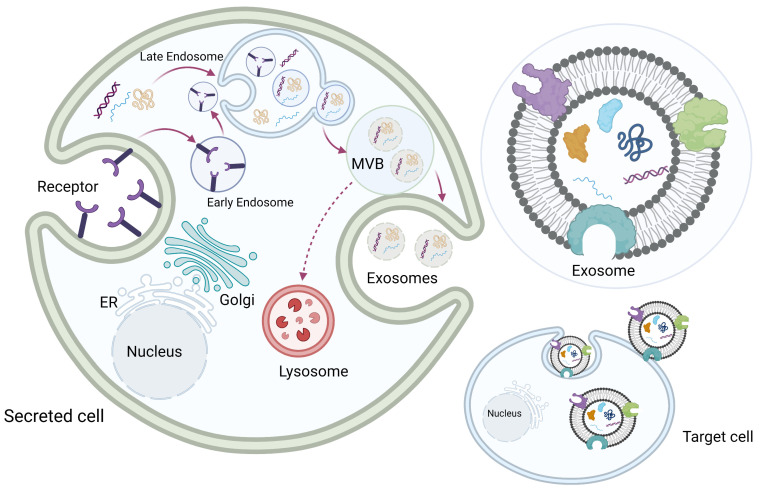
Exosome biogenesis. Exosome biogenesis begins with invagination of the cellular plasma membrane to form early endosomes. Early endosomes then mature into late endosomes, and the invagination of the endosome membrane forms multiple intraluminal vesicles inside the endosome, which becomes a multivesicular body (MVB). Each intraluminal vesicle inside the MVB encloses part of the cytosol, including various proteins and nucleic acids. The MVBs then fuse with the plasma membrane, and exosomes are released into the extracellular space. Some MVBs fuse with the lysosomes to be degraded. Original photo created with BioRender.com.

Exosomes originate from the multivesicular late endosome. Exosome biogenesis begins with the invagination of the cellular plasma membrane to form early endosomes. Early endosomes then mature into late endosomes, and the invagination of the endosome membrane forms multiple intraluminal vesicles inside the endosome, which becomes a multivesicular body (MVB). Each intraluminal vesicle inside the MVB encloses part of the cytosol, including various proteins and nucleic acids. The MVBs then fuse with the plasma membrane, and the exosomes are released into the extracellular space. Some MVBs fuse with the lysosomes to be degraded (Figure 2) [8,23,24,25,26].

### 2.3. Exosomes Content

The released exosomes express on their surface biological markers and contain a variety of bioactive substances including lipids, proteins, enzymes. Some of these constituents are common between all exosomes; however, evidence suggests that exosome contents change significantly based on the body’s status in health and disease [8,23,27,28].

Exosomes are highly enriched in proteins and lipids such as cholesterol, glycosphingolipid, phosphatidylserine, and ceramide. Lipids have an essential role in supporting the shape of exosomes, encouraging biogenesis, and regulating homeostasis. lysobisphosphatidic acid (LBPA) is a high-density lipid that is present in the internal membrane of MVBs and helps in exosome formation by interacting with Alix protein to facilitate the inward sprouting of the membrane of MVBs. Other lipid classes contained are bismonoacylglycerol phosphate (BMP), sphingomyelin and phosphatidylcholine [28]. Exosomes are also known to have a high concentration of sphingomyelin compared to other extracellular vesicles. Studies have shown that exosomes could alter the lipid composition of recipient cells when they are transferred to target cells, particularly cholesterol and sphingomyelin, to achieve cell homeostatic functions [8,28].

Exosomes also carry different RNA moieties with variable lengths and are considered as gene expression regulators. Exosomes contain messenger RNA (mRNA), microRNA (miRNA), small nuclear RNAs (snRNAs), small nucleolar RNAs (snoRNAs), transfer RNAs (tRNAs), ribosomal RNAs (rRNAs) and long noncoding RNA (lncRNA) [26,27,29]. In addition to RNAs, different types of DNA have been detected in exosomes; single-stranded DNA, double-stranded DNA and mitochondrial DNA. DNA-incorporated exosomes can be transferred to target cells to exert functional consequences [27].

In addition, exosomes are enriched with endosomal sorting complexes required for transport (ESCRT), such as TSG101 and CHMP4. ESCRT contributes to exosome biogenesis and secretion where it mediates MVB formation and vesicle budding [8,27,30]. Additionally, along with ESCRT, the Alix protein has been reported to participate in inward budding and cargo selection [31].

The released exosomes also express on their surface biological markers that help in exosome identification and can be used as diagnostic biomarkers and/or disease progression indicators. These proteins are categorized into integrins, CAMs, tetraspanins, and MHC class I and II [8,23,32].

## 3. Exosomes Transport through the Blood–Brain Barrier

Passive diffusion and active transport help to selectively transport substances through the BBB. The mode of transport depends on the physical and chemical properties of the substances to be transported, including molecular weight, lipid solubility, plasma protein binding, and cerebral blood flow. Passive transport is limited to small-sized molecules that have sufficient lipid solubility such as gaseous oxygen and carbon dioxide molecules. On the other hand, substances that cannot cross the BBB through passive transport require an active transport system, including carrier-mediated transporters, efflux pumps, receptor-mediated transporters, and adsorption-mediated transcytosis [13,33,34,35,36,37].

Exosomes have been shown to cross the BBB from the blood to the brain, and vice versa [29]. However, the mechanistic ways of how exosomes cross from peripheral circulation to the brain have not yet been fully elucidated [29]. A large body of evidence suggests that exosome transport through the BBB occurs primarily through transcytosis—similarly to immune cells and infectious agents. Therefore, unlike the paracellular pathways that allow brain cells to cross the extracellular space, through transcytosis, exosomes cross the intracellular compartment [29]. Nevertheless, studies speculate two possibilities for the uptake of exosomes by the brain, either by complete passage through the endothelial cell barrier or by being sequestered inside the brain endothelial cells. The former helps exosomes exert their effects on the entire brain, whereas sequestration affects the brain endothelial cells, which results in regulated and specific transport mechanisms [19,29,38].

**Figure 3:**
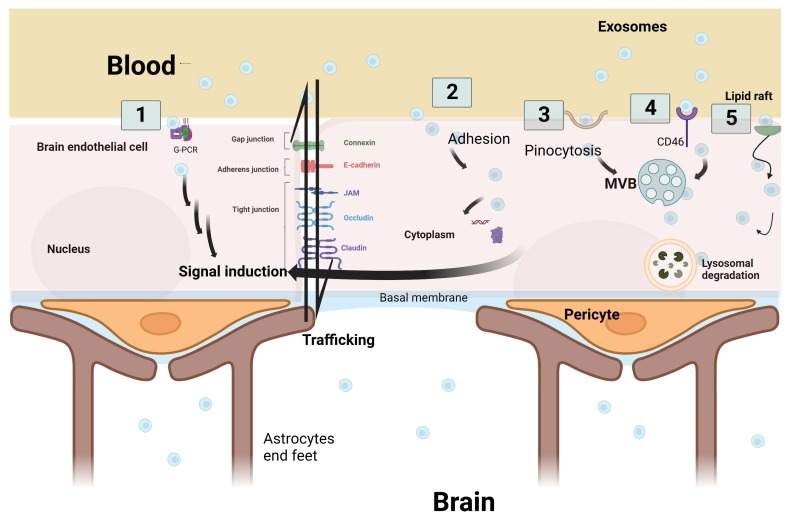
Schematic representation of the five hypothetical routes of exosome transcytosis through the brain endothelium at blood–brain barrier. (1) Exosomes from the blood bind to G-protein coupled receptor (GPCR) at the brain endothelium and induce downstream signaling cascade pathways. (2) Adhesion of exosomes and fusion through the endothelial cell membrane is followed by the secretion of exosome cargo into the cytoplasm to induce signal cascades. (3) Exosomes are incorporated into the cells by inverse membrane invagination. (4) Exosomes from the blood bind to receptors such as CD46, which leads to exosome entry into the cell. (5) Non-specific lipid raft that binds to exosomes and aids in their incorporation. Three outcomes are expected after transcytosis: signal induction, lysosomal degradation, or trafficking through brain endothelial cells. Original Photo created with BioRender.com.

According to Julien Saint-Pol et al., five hypothetical pathways are suggested to explain the communication and interaction between exosomes and the receiving target brain cells: (1) binding of exosomes onto the cell surface with a G protein-coupled receptor (GPCR) to induce signaling cascade pathways; (2) internalization of exosomes by adhesion to the cell membrane and fusion to release the exosomes’ intracellular components inside the cellular cytoplasm—leading to a series of intracytoplasmic events; (3) micropinocytosis, which is the incorporation of exosomes into the cells by inverse membrane invagination to form a vesicle within the brain cells; (4) receptor-mediated transcytosis that leads to the exosomes’ entry into the cell through endocytosis and its storage in the MVB; or (5) nonspecific lipid rafts that influence membrane fluidity, exosome trafficking and thus regulate neurotransmission. Three outcomes are expected after exosome passage into brain cells: lysosomal degradation, cell signal induction due to content release into the cytosol, or neoformed ILVs in the receiving cell due to transfer from the MVB to the plasma membrane (Figure 3) [4,39].

In order to study the entry mechanisms, labeling exosomes with fluorescent agents such as PHK26 and DiD can be useful, which can in turn reflect the physiological status of the brain and the integrity of the BBB [40,41]. In line with this, a study was able to report the initial entry steps of labeled exosomes, starting with the physical contact of barrier fusion; exosomes interact and fuse with barrier-type endothelial cells to release their cargo into the cytoplasm. The following steps observed are referred to as paracytosis and transcytosis [42,43]. However, the molecular mechanisms of transcytosis may depend on the size and density of the exosomes [44,45]. In a recent study, electron microscopy revealed that the low-density exosome subpopulations are found in the luminal side of endothelial cells, whereas high-density subsets accumulate in the abluminal side [44,45].

### 3.1. Regulation of Transcytosis

The molecular mechanisms of transcytosis are influenced by several regulators involving Rab-recycling endosomes, the Rab family of small GTPase and EEA1 [46]. The latter is an early endosome antigen 1 that has a crucial role in the trafficking and distribution of endocytic proteins, allowing them to fuse with membrane proteins by binding to phosphatidylinositol-3-phosphate via the C-terminal domain. Evidence shows that SNAREs are important in the fusion of vesicles to the plasma membrane, particularly by mediating vesicle fusion in exocytosis [22]. Rab 11 recycling endosomes are also contributors of transcytosis regulation, as they deliver the cargo of exosomes to the basolateral membrane and mediate exocytosis. Rab 11 depletion leads to the accumulation of recycling endosomes that include transferrin receptors [22,47]. Any changes in the function of some Rab GTPases could also affect biogenesis since the alterations could facilitate the fusion of late endosomes with basolateral membranes [48].

Studies have reported that lipopolysaccharide (LPS), Wheatgerm agglutinin (WGA), and mannose 6-phosphate (M6P) influence the passage of exosomes through the BBB via different mechanisms [29]. For instance, LPS enhances adsorptive transcytosis and activates the transportation of immune cells by stimulating cytokine release. LPS also affects prostaglandin-dependent pathways, leading to BBB disruption [49,50]. WGA promotes glycoprotein adsorptive transcytosis, particularly glycoproteins that contain *N*-acetyl-D-glucosamine and sialic acid [29], while substances that bind to the M6P receptor are inhibited from trafficking across the BBB [51]. Banks et al. tested the effects of LPS, WGA and M6P on exosome transport across the BBB. They found that LPS enhances exosome uptake in a complex manner, and they attributed this to the previously mentioned mechanisms. WGA and M6P exert effects depending on the molecules and the receptor binding site, respectively (Table 1) [29].

### 3.2. Exosomes Transport through the BBB during Inflammatory Conditions

Inflammatory conditions can enhance exosome trafficking across the BBB. This is because inflammation disrupts the BBB and enhances vesicular penetration, where exosomes are absorbed by brain microvascular endothelial cells through endocytosis [29]. In this context, evidence indicates that following hypoxic conditions, massive amounts of intracellular calcium are released into the cytoplasm that participate in the disruption of the BBB, particularly through the alteration of junctional proteins, eventually leading to a huge influx of exosomes into the BBB [52]. As a result, disruption of the BBB and the continuous leakage of exosomes could make exosomes real-time biomarkers for early diagnosis and to monitor the progression of inflammatory conditions [53]. Supporting this notion, exosomes harbor inflammatory signals such as α-synuclein and prions that contribute to the progression of neurodegenerative diseases. Therefore, exosomes could be exploited as potential biomarkers that can help in the early detection of brain disease [54]. As exosomes are secreted by different types of cells and contain a wealth of cell components reflecting the physiological status of the cell, such as proteins, lipids, metabolites, and RNAs, they represent important tools for the identification of disease biomarkers. However, the use of exosomes to identify biomarkers can be challenging because of the difficulty in exosome isolation and the low yield that can be challenging to test using the proteomic analytical techniques insensitive to the low amounts of material tested [55].

Brandon Dow Chan et al. suggested that brain microvascular endothelial cells (BMECs) must be preconditioned with inflammatory mediators such as interleukin 6 (IL-6) and tumor necrosis factor alpha (TNF-alpha) to allow for endocytosis of exosomes [56]. Other studies support this theory and explain how inflammation promotes the transport of exosomes during metastasis of cancer cells [38,56,57]. Increasing evidence has explained this phenomenon in the context of the combination between exosomes that contain metalloproteinases, TNF-alpha, IL 1-β and interferon γ as inflammatory mediators to promote degradation of the extracellular matrix and that disturb the BBB [56,57,58,59].

Exosomes can enter the brain similar to viruses through receptor-mediated micropinocytosis or endocytosis. This can be explained due to their size, which resembles the virus’s nanosize [26,38,56,57,60]. As per Grapp et al., the interaction between the receptor membrane and the exosome’s surface folate receptor-α leads to exosome permeation and entry to the brain parenchyma from the CSF [61,62,63].

Chen et al. elucidated how exosomes cross the BBB in an in vitro model. Genetically engineered cell-derived exosomes were used with in vitro BMECs to compare the transport of these exosomes in normal status and stroke as neuroinflammatory condition. Chen et al. found that cell-derived exosomes bypass the BBB under stroke-like conditions more often as compared to healthy conditions by utilizing active BMEC endocytosis, particularly receptor-mediated endocytosis, lipid raft-mediated endocytosis, and macropinocytosis [64].

### 3.3. Exosomes Transport through the BBB during Brain Metastasis

Cancer cells produce an abundance of exosomes that play a role in cancer cell migration, invasion, and metastasis through angiogenesis. Tumor-cell-derived exosomes can interact with the vasculature tissues, including the brain vasculature, predisposing those tissues to angiogenic factors and metastasis. Additionally, exosomes derived from cancer cells carry immunosuppressive proteins that suppress immune system functions and that promote tumor growth [65,66,67,68,69]. Emerging evidence reports that lncRNAs are involved in the progression of cancer via different mechanisms [70]. Yunhe Lu et al. reported that exosomes derived from triple negative breast cancer cells have high metastatic capacity in the brain, as these exosomes carry the lncRNA gene GS1-600G8.5, which helps in disrupting the BBB and enhances its permeability, thus promoting metastasis [71].

In addition, exosomes influence the tumor microenvironment to promote metastasis since they mediate cell–cell communication. Li et al. reported that exosomes secreted by cancerous cells enhance tumor progression and increase chemotherapy resistance by affecting the tumor environment and the oncogenic pathways. Their study also highlighted the key role of exosomes in configuring organs as pre-metastatic niches for future metastasis. For instance, exosomes secreted from breast cancer cell lines contain functional miRNA that is able to alter the BBB and that contributes to brain metastasis [72]. In this context, alterations in the BBB induced by microRNAs have been evidenced, and more research is still needed to better understand the effect of microRNAs carried by the exosomes through the BBB in general and in the context of brain metastasis in particular [73,74,75,76,77,78,79,80].

Morad et al. showed that tumor-derived EVs can breach the BBB through transcytosis. Their study examined the effects of EVs derived from triple negative breast cancer cells in promoting brain metastasis, and they explored the mechanisms of crossing the BBB. It was found that some subpopulations of EVs disrupt the BBB by altering the inter-endothelial tight junctions. However, their study did not specify whether these subpopulations contain exosomes or not. Furthermore, the study showed that the mechanistic steps of transcytosis are reliant on the clathrin-dependent pathway and not the caveolin-dependent pathway, which involves macropinocytosis in the uptake of EVs by brain endothelial cells. It was also reported that these extracellular vesicles regulate their transcytosis, particularly by facilitating the late endosomal pathway through downregulation of the late endosomal marker rab7 [81].

## 4. Novel Therapeutic Approaches of Exosomes

### 4.1. Advantages of Therapeutic Exosomes in Drug Delivery

**Figure 4:**
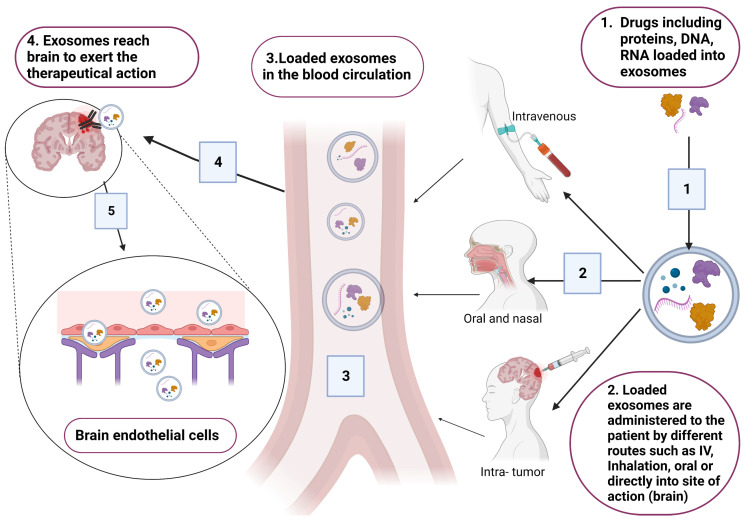
Schematic presentation for the incorporation of different therapeutical drugs, including DNA, RNA and proteins. Loaded exosomes are administered to patients based on the site of action (brain). Suitable routes that have good bioavailability are the intravenous route through injections, oral or inhalation, and intra-tumor injection. Original photo created with BioRender.com.

Exosomes are considered as successful delivery systems that transfer drugs and genes to target cells. In the context of brain diseases, exosomes are considered as good tools that could be used for treating brain diseases due to their ability to cross the BBB [4,39]. Common exosomes tested for delivery are synthetically engineered exosomes that are encapsulated with therapeutical agents (Figure 4). However, exosomes loaded with endogenous molecules and exosome-based immunotherapy are also under investigation for treating certain CNS pathologies [61,82,83].

In recent years, artificially engineered exosomes have been developed as better alternatives to natural exosomes in terms of large-scale production, standardized isolation, drug encapsulation, stability and quality assurance. Manufactured exosomes are considered as potentially effective carriers for chemical and biological therapeutics, as we can control circulation time and selectivity [84]. Transition strategies regarding synthetic exosomes have appeared to overcome the limitations of endogenously produced exosomes [84,85,86]. However, therapeutic exosomes in general compared to other nanocarriers have several advantages, including specific targeting, safety profile, histocompatibility, bioavailability, and administration. These characteristics permit the directed delivery of exosomes to the disease site, as they can bind to assigned receptors effectively, resulting in precise individualized treatment [87]. Exosomes are considered as ideal biomimetic vehicles for endogenously loaded molecules such as RNA-based therapies. This cargo can be specifically loaded into the donor cells to regulate the function of the recipient cells [88,89].

On the other hand, biological therapies can be delivered to the targeted cells naked without any molecular conjugation or vector. In this context, gymnosis has emerged as a process to deliver antisense oligonucleotides without transfection. Souleimanian et al. showed that gymnotic silencing is effective in targeting the BCL-2 protein in treating B-cell leukemias and lymphomas [90]. However, exosomes are considered as ideal carriers for antisense oligonucleotides, including messenger RNAs, as they provide protection against the immune system, lysosomal degradation, and instability issues [91]. Recently, the area of encapsulation of molecular therapeutics in exosomes has risen to overcome the key hurdles of gymnosis [91,92].

It has been established that exosomes have a long half-life compared to other nanocarriers such as liposomes and micelles (Table 2). This can be attributed to their nanosize and the presence of surface CD47 that both help in evading macrophages and monocytes. Consequently, exosomes are considered as a relatively safe drug-delivery system, as they are able to overcome immunogenicity issues, a limiting factor reducing the effectiveness of other nanocarriers [93,94,95].

Extracellular vesicles (EVs), including exosomes, have been shown to play a role in the delivery of functional mRNAs using lipid nanoparticles (LNPs). Nawaz et al. delivered VEGF-A mRNA via LNPs and studied how the transport of LNP mRNAs between cells is extended by the cells’ EVs. The delivery of therapeutic VEGF-A mRNA to ischemic tissues for producing new blood vessels is an emerging strategy for the treatment of cardiovascular diseases. The results show that cellular uptake of LNPs and their mRNA molecules occurs quickly and that that LNPs transform EVs as functional extensions to distribute therapeutic mRNA between cells, where EVs deliver this mRNA differently than LNPs [96].

**Table 2:** The advantages and disadvantages of lipid nanosized vehicles and exosomes.

Lipid Nano Carrier	Advantages	Disadvantages	Reference
Liposomes	Large-scale production, biodegradable, carry hydrophilic and hydrophobic drugs.	Serum proteins can bind to the unmodified surface, less stable and lower blood circulation time.	[97]
Nanomicelles	Carry hydrophilic and hydrophobic drugs, lower critical micelle concentration, biodegradation and improved solubility.	Low encapsulation efficacy, lower stability, and insufficient cellular interaction.	[98,99]
Exosomes	More stable, biocompatible, long-term safety, highly selective and able to escape the host immune system.	Difficult in isolation and preparation on a large scale, might promote cancers and induce oncogenic pathways.	[95,100,101]

### 4.2. Potential Therapeutical Applications of Exosomes in Treating CNS Diseases

According to recent evidence, exosomes derived from cerebral cells carry regulatory elements and transport these regulatory elements to the injury sites in the brain to aid in tissue regeneration [18]. Similarly, exosomes derived from stem cells (SC) are considered as a novel therapeutic approach for traumatic brain injury (TBI) via regulation of post-transcriptional genes in recipient cells. This goal was met in a proof-of-concept study in which MSC-derived exosomes were given intravenously to adult rats who were subjected to TBI, which resulted in enhanced neuroplasticity and functional recovery. This may be due to the expression of miR-133b, which is contained in the exosomes. The advantages of SC-derived exosomes are reported in this study as being effective, stable and having a long shelf life [102,103,104]. Exosomes secreted from stem cells mimic the derived cells’ phenotypes. The exchange of genetic information via intercellular communication mediated by stem cells and exosomes has the potential to enhance tissue regeneration and differentiation. Exosomes derived from stem cells are regarded as evolving communication factors where they cause persistent genomic reprogramming in the receiving cells to control their fates [105]. However, to fully understand the mechanism and regenerative potential of SC-derived exosomes for the treatment of neural injuries and neurodegenerative diseases, additional research is needed [106]. Moreover, exosomes derived from multipotent mesenchymal stromal cells facilitate angiogenesis, remodeling, and neurogenesis in stroke patients. A randomized single-blinded, placebo-controlled clinical trial has been conducted to evaluate the safety and effectiveness of miR-124-enriched exosomes to alleviate brain injury during cerebrovascular diseases [82,107]. As previously mentioned, synthetic or engineered exosomes can be used to encapsulate therapeutic drugs [7,108]. Clinical trials are now evaluating the delivery of exosomes loaded by anti-inflammatory agents and growth factors for patients with anxiety disorders, refractory depression, and neurodegenerative diseases, through ultrasound as a noninvasive method to reversibly and transiently increase permeability of the BBB, enhance blood flow, and thus deliver exosomes through the BBB [61,82,109].

Furthermore, exosomes can play an effective role in developing personalized medicine to treat malignant tumors, mental disorders, neurodegenerative diseases by incorporating different RNA subtypes [19,110]. Small interfering RNA (siRNA) could be used for specific knockdown of genes without triggering the immune system response. When siRNA-loaded extracellular vesicles were transfected by sonication, silencing of HER 2 oncogene in breast cancer was induced by 50%, which proves their functionality in recipient cells [93,96].

MicroRNA can be loaded into exosomes endogenously. Accumulating evidence indicates that exosomes carry miRNAs, that regulate many genes and influence one or more cellular pathways in target cells [111]. Expression or attenuation of the specific miRNAs mediated by exosomes have a fundamental role in the treatment of diseases [64]. Exosomal miR-193b, for example, suppresses the expression of neuronal amyloid precursor protein, reducing the progression of Alzheimer’s disease [111]. In addition, neurons can produce exosomes loaded with miR-124a, which in turn control astrocytic glutamate that is crucial in synaptic transmission regulation [108,112]. However, the use of miRNA cargo is limited due to its short half-life and peripheral accumulation in the liver and kidneys. In this context, cell membrane-cloaked nanoparticles and exosome-mimics have been designed as vectors for delivering targeted miRNA and other drugs with higher efficiency [108].

Fusing target therapeutic proteins with one of the exosome’s basic constitutive proteins also aids in increasing the concentration of the target protein in the targeted cells. However, the impact of constitutive proteins on the function of targeted proteins as well as technical difficulties in exosome purification and processing limit this method, resulting in slow progress in clinical trials. SCAMP 5 is a protein that is secreted by the membrane of exosomes and is a member of a family that has a pivotal role in targeting and sorting exosomes. This protein is preferentially localized to mediate clearance and to remove aggregation of the alpha-synuclein toxin in the brain of patients with neurodegenerative diseases such as Huntington’s disease [110,113].

In brain cancer, a phase I clinical trial was conducted to emphasize the effect of a novel antisense molecule (IGF-1R/AS ODN) in treating malignant gliomas to suppress oncogenes and to kill tumor cells by apoptosis. According to this pilot study, the presence of the antisense molecule with exosomes stimulated the adaptive immune system against tumor cells as an effective immunotherapeutic vaccine to decrease brain glioma with a reasonable safety profile [82,114]. Shi et al. found that conjugation between modified genetically engineered exosomes and other chemotherapeutic drugs such as paclitaxel and temozolomide yield a better curative efficacy against brain tumors in comparison to single-agent therapy [61]. Furthermore, Ha et al. reported that using zebrafish as a brain cancer model treated with exosome-loaded doxorubicin showed significant therapeutic efficacy compared to doxorubicin alone [101].

Furthermore, multiple studies show that natural killer (NK)-cell-derived exosomes can be a potent and less toxic targeted immunotherapy toward glioma compared to conventional chemotherapy and radiotherapy [115]. Nonetheless, the underlying antitumor mechanism is still not fully elucidated. According to Hao et al., exosomes that are produced from human umbilical cord-derived mesenchymal stem cells have significant anti-cancer activity in the early glioma stage through regulation of the miR-10a-5p/PTEN signaling pathway [61,116].

In the field of regenerative medicine, stem cells are of interest, as they are non-immunogenic and noninvasive compared to normal transplantation procedures. Recent promising therapeutic approaches utilize stem-cell-derived exosomes to stimulate endogenous neural progenitors against a variety of cerebrovascular diseases [69]. Ghosh et al. found that exosomes derived from human-induced pluripotent stem cells (hiPSCs) and mesenchymal stem cells (MSCs) might be considered as promising tools to treat the post-traumatic brain through the interaction of exosomes and brain parenchyma cells with the neurogenic niche, resulting in brain remodeling and neurogenesis [117].

Additionally, exosomes can help in the treatment of multiple sclerosis (MS). A research study found that exosome-based stem cell therapy has the potential to treat MS, as it enhances motor and neural function while reducing myelin loss and neuroinflammation [118]. In a comparable study, an animal model of MS recovered after receiving intranasal curcumin-based exosomes, which demonstrated the ability of exosomes to bypass the BBB and exert therapeutic effects in the brain [119].

The use of macrophage-derived exosomes as a novel therapeutic modality for Parkinson’s disease (PD) has also been reported. Catalase, a potent antioxidant, has been incorporated into exosomes ex vivo to successfully achieve substantial neuroprotection in vitro and in vivo on a PD model (Table 3) [119].

### 4.3. Challenges and Limitations of Exosomal Therapy

Despite the aforementioned features of therapeutical exosomes, there are some challenges hurdling this therapeutical approach, ranging from the expensive and low yielded purification techniques to the need of effective large-scale production for clinical use [101]. That being said, the large load of exosomes could also affect the stability of exosome formulation through aggregation, leading to administration difficulties [120]. Moreover, the internal composition of exosomes themselves might be a critical limitation, as the cargo can contain heterogenous molecules that could trigger immunogenic reactions [101].

Another important concern is that exosomes could facilitate oncogenic pathways or decrease the sensitivity of anticancer agents, leading to tumor dissemination and metastasis [101]. For instance, representing cellular stress, exohypoxia or exosomes produced in hypoxic conditions serve as a crucial factor for cancer development and subsequent invasion and metastasis [121,122].

## 5. Clinical and Preclinical Cases Related to CNS

Although clinical use of exosomes is still in its early stages, there has been a surge of interest in clinical trials involving exosomes in a variety of pathologies. Exosomes tested in clinical trials are mainly derived from two sources: human-specimen-derived exosomes or plant-cell-derived exosomes. In CNS-related clinical trials, exosomes are being used as biomarkers, cell-free therapy, and as a form of cancer immunotherapy [123].

As stated earlier, the presence of exosomes in many bodily fluids as earned them recognition as disease biomarkers. Preclinical studies have validated the possibility of exosome use in disease diagnosis [124]. In the case of glioma, Shi et al. demonstrated that a higher level of exosomal miR-21 is present in the cerebrospinal fluid of glioma patients compared to healthy individuals and suggested miR-21 as a potential glioma biomarker [125]. Three of the four clinical trials evaluating exosomes as biomarkers for central nervous system (CNS)-related diseases listed in ClinicalTrials.gov are currently recruiting participants. The clinical trial NCT05035134 seeks to utilize circulating exosomes in monitoring patients with intracerebral hemorrhage, while the second, NCT05370105, aims to identify exosomes as new biomarkers for profiling stroke patients prior to and after rehabilitation. Additionally, RNA and protein exosomal cargo in TBI patients is being used to develop a diagnostic signature in the clinical trial NCT04928534. Researchers at the University of Alabama at Birmingham have completed a clinical trial in patients with Parkinson’s disease (NCT01860118). The trial explored exosomal biomarkers associated with the progression of the disease, but the results are still pending [82].

In a series of preclinical studies, exosomes have shown promise as a therapeutic tool [82,124,126]. A research paper has demonstrated that functional recovery after stroke in rat models was aided by exosomes isolated from the serum of healthy rats [127]. All exosome-based therapy clinical trials for CNS diseases are currently uncompleted. The ability of MSC-derived exosomes to reduce disability after an acute ischemic stroke is being investigated in a phase I and II clinical trial (NCT03384433). Another clinical trial (NCT05326724) aims to examine the potential therapeutic use of acupuncture-induced exosomes in post-stroke dementia. Finally, scientists from China’s Ruijin Hospital are conducting a phase I and II clinical trial (NCT04388982) to evaluate the safety and potential of MSC-derived exosomes in treating Alzheimer’s disease [82].

Exosome-based immunotherapy has had promising outcomes against a wide range of diseases [128]. A completed phase I clinical trial (NCT01550523) has proposed the use of exosomes in glioma patients, containing tumor antigens released from the tumor itself upon apoptosis, to be used as an immunotherapeutic tool to activate the patient’s immune system, but the results have not been published by the researchers [82].

## 6. Exosomes Patents

Extensive study in the realm of exosomes has produced numerous novelties and intellectual properties. Patent certifications have been awarded for innovations related to exosome production, isolation, transport, and therapeutic application [129].

Therapeutic patents are aimed to improve exosome purification, localization, and selective targeting. Most assets beyond this approach focus on surface-engineered exosomes that overexpress specific biological molecules. For instance, Evox Therapeutics granted a novel perspective to protect exosomes carrying biological molecules such as DNAs and RNAs via nucleic acid binding proteins PUF, Cas6, Cas13 or the nucleic acid aptamer binding protein [130].

Similarly, Kevin P. Dooley et al. generated surface-engineered exosomes that express abundant membrane proteins to provide desired therapeutic effects [131].

Raghu Kalluri et al. also invented exosomes that have CD47 on their surface to be ready for therapeutic agent incorporation [132]. A novel invention has been used to promote myelination in neurodegenerative disorders, such as multiple sclerosis (MS), and other neurological disorders associated with demyelination by using exosomes as lipid nanosized vesicles loaded with specific nucleic acids that can be modified with surface proteins identified in these exosomes [133].

Exosomes acting on tissue regeneration and healing have been invented by Eduardo Marbán et al. in this aspect; exosomes are synthesized from a specific cell type and are loaded with proteins to target the injured tissue [134].

Other novel exosome-based formulations have been certified with aims to promote biological agent delivery by crossing the BBB and by targeting inflammatory tissues and tumors, hence increasing the therapeutic benefits while reducing an immune reaction to the agents. Another feature of this invention is the technique of exosome composition for delivering functional polynucleotides that are not naturally present in exosomes [135]. We have clearly begun to comprehend the fundamentals and principles of exosomes. However, further research is required to develop more patents in targeting and therapeutics (Table 4).

## 7. Conclusions and Way Forward

Compelling evidence points to the unique ability for bi-directional transfer of exosomes through the BBB and thus their important role in normal physiological functions of the CNS as well as their contribution to disease pathogenesis and progression. Importantly, exosomes have the potential to transport drugs through the BBB and then into the targeted cells with minimal immunogenicity. However, parameters such as lipophilicity, concentration gradient, enzymatic activity, and clearance kinetics, in addition to illness induction and modulation, have an impact on BBB penetration and exosome permeability. We assume to overcome the aforementioned limitations and challenges of exosomes in the future to successfully deliver therapeutic agents to targeted brain tissues through the BBB.

Therefore, a better understanding of how exosomes cross the BBB, and their mechanisms of action and effect, will help to design and develop novel therapeutics for the treatment of brain diseases. Future studies will help in engineering optimized exosomes enclosed with drugs or in carrying genetic material effective in crossing the BBB, allowing for individualized therapy to treat brain diseases. Further investigations and studies focusing on the challenges associated with therapeutic dose, route of administration and other pharmacokinetic parameters of exosomes are also needed to prevent off-site targets and adverse effects.

## Figures and Tables

**Table 1:** Summary of molecular regulators of transcytosis and their roles.

Molecular Regulator of Transcytosis	Role in Transcytosis	Reference
Rab recycling endosomes (EEA1; Rab 11a; Rab 11b)	Trafficking and distribution of endocytic proteins, allowing them to fuse with membrane proteins.	[22,46,47]
	Delivery of the cargo of exosomes to the basolateral membrane and mediate exocytosis.	
SNAREs	Fusion of vesicles to the plasma membrane.	[22]
LPS	Adsorptive transcytosis and activation of transportation of immune cells.	[49,50]
WGA	WGA promotes glycoproteins adsorptive transcytosis.	[29]
M6P	Substances that bind to the M6P receptor are inhibited from trafficking across the BBB.	[51]

**Table 3:** Therapeutical applications using exosomes.

Exosome Type\Exosomal Content	Mechanism	Therapeutical Application	References
Exosomes derived from cerebral cells	Carry and transport regulatory elements to the injury sites in the brain	Tissue regeneration	[18]
Exosomes derived from stem cells	Regulation of post-transcriptional genes in recipient cells	Traumatic brain injury (TBI)	[102,103,104]
Exosomes derived from multipotent mesenchymal stromal cells	Facilitate angiogenesis, remodeling, and neurogenesis	Stroke	[82,107]
Exosome-loaded drugs	Load exosomes with anti-inflammatory agents and growth factors	Anxiety disorders and refractory depression	[61,82,109]
Exosomal miR-193b	Suppresses the expression of neuronal amyloid precursor protein	Alzheimer’s disease	[111]
Exosomes derived from neurons	Control astrocytic glutamate	Synaptic transmission regulation	[108,112]
SCAMP 5	Mediates clearance and removes aggregation of alpha-synuclein toxin	Huntington’s disease	[110,113]
Antisense molecule with exosomes	Stimulate the adaptive immune system	Brain glioma	[82,114]
Natural killer (NK)-cell-derived exosomes	Immunotherapy	Glioma	[115]
Exosomes of human umbilical cord-derived mesenchymal stem cells	Regulate miR-10a-5p/PTEN signaling pathway	Early glioma stage	[61,116]
Stem-cell-derived exosomes	Stimulate endogenous neural progenitor	Cerebrovascular diseases	[68]
Exosome-based stem cell therapy	Enhances motor and neural function while reducing myelin loss and the neuroinflammation	Multiple sclerosis (MS)	[118]
Macrophage-derived exosomes	Neuroprotection	Parkinson’s disease (PD)	[119]

**Table 4:** List of some therapeutic exosome patents.

Patent Name	Patent Number	Reference
Use of exosomes for the treatment of disease	WO2016201323A1	[132]
Exosome-based therapeutics against neurodegenerative disorders	US11369634B2	[133]
Exosomes and microribonucleic acids for tissue regeneration	US11220687B2	[134]
Biological agent–exosome compositions and uses thereof	US11458097B2	[135]

## Data Availability

Not applicable.
